# Age, geographical and socio-economic related inequalities in contraceptive prevalence: evidence from the 1993–2014 Ghana Demographic and Health Surveys

**DOI:** 10.1186/s40834-022-00194-9

**Published:** 2023-02-07

**Authors:** Felix Mensah, Joshua Okyere, Simon Agongo Azure, Eugene Budu, Edward Kwabena Ameyaw, Abdul-Aziz Seidu, Bright Opoku Ahinkorah

**Affiliations:** 1 Department of Data Science and Economic Policy, University of Cape Coast, Cape Coast, Ghana; 2 Department of Population and Health, University of Cape Coast, Cape Coast, Ghana; 3 Department of Nursing, College of Health Sciences, Kwame Nkrumah University of Science and Technology, Kumasi, Ghana; 4 School of Public Health, Population and Reproductive Health Division, University of Port Harcourt, Choba, Nigeria; 5 Korle Bu Teaching Hospital, P. O. Box, 77, Accra, Ghana; 6 L & E Research Consult Ltd., Wa, Upper West Region Ghana; 7 Institute of Policy Studies and School of Graduate Studies, Lingnan University, Tuen Mun, New Territories, Hong Kong; 8 REMS Consult LTD, Takoradi, Ghana; 9 College of Public Health, Medical and Veterinary Sciences, James Cook University, Townsville, Australia; 10 School of Public Health, Faculty of Health, University of Technology Sydney, Sydney, Australia

**Keywords:** Contraception, Demographic and Health Surveys, Ghana, Global health, Inequality

## Abstract

**Background:**

Contraceptives afford individuals the opportunity to meet their reproductive needs and reduce maternal mortality. We aimed at assessing the trend and inequalities of contraceptive use in Ghana based on the 1993–2014 Ghana Demographic and Health Surveys.

**Methods:**

We used the World Health Organization’s Health Equity Assessment Toolkit (HEAT) software in analysing the data. We adopted two approaches for the analysis. First, we disaggregated inequalities in contraceptive use using four equity stratifiers: wealth index, education, residence, and region. Second, summary measures (D), (PAR), (R), and (PAF) were also employed. A 95% uncertainty interval (UI) was constructed for point estimates to measure statistical significance.

**Results:**

Contraceptive prevalence increased from 20.3% in 1993 to 26.7% in 2014. The contraceptive prevalence among women aged 20–49 increased from 20.6% [95% UI = 19.1, 22.3] in 1993 to 26.8% [95% UI = 24.9, 28.9] in 2014 and this exceeded the increase that was recorded among those aged 15–19 (from 13% [95 UI = 8.7, 19] to 18% [95% UI = 11.5, 28.6]), in the same period. It was evident that substantial inequality existed with respect to contraceptive use, from 1993 to 2014, with widest inequality occurring in 2003 (PAF = 2.7, 95% UI = -16.6–21.9; D = 17.4, 95% UI = 12.7–22.1). In terms of wealth index, the least inequality was observed in 2014 (PAR = 1.3, 95% UI = -1–3.6; D = 5.9, 95% UI = -0.1–12). Regarding education, the widest inequality occurred in 1993 (PAF = 138.6, UI = 132.1–145.1; D = 40.1, 95% UI = 34.4–45.9). With place of residence, the widest gap in inequality occurred in 1993 (PAF = 51.2, 95% UI = 46.2–56.3; D = 15.3, 95% UI = 11.8–18.7). There was inequality in contraceptive use with respect to sub-national regions. In 2014, the Difference (D = 21, 95% UI = 14.6–27.4) and the PAF (PAF = 20.9, 95% UI = 11.2 − 30.5) measures revealed substantial absolute and relative regional inequality between the regions.

**Conclusion:**

There was a steady increase in contraceptive use from 20.3% in 1993 to 26.7% in 2014. Nevertheless, the percentage change is minimal. The trends of inequality indicate that inequalities in contraceptive use was evident across the dimension of age, place of residence, wealth index, education, and region. Yet, there was a substantial reduction in inequalities related to contraceptive use in 2014. Therefore, targeting adolescents, women in rural areas, low wealth quintile, and those with no formal education is key to substantially improving contraceptive use across the country.

## Introduction

Contraceptive use affords individuals the opportunity to meet their reproductive needs and reduce maternal mortality [1]. These contraceptives include modern and traditional methods [2]. According to Hubacher and Trussell [3], the modern contraceptive method is a product or medical procedure that interferes with reproduction. Modern methods of contraceptives include; sterilization, intrauterine devices, subdermal implants, oral contraceptives, condoms, injectables, emergency contraceptive pills among others [3]. Aside modern contraceptive methods use, there are traditional methods or non-modern methods that are available to persons in meeting their contraception needs. These traditional methods include; withdrawal, lactational amenorrhea, abstinence, and fertility awareness approaches [3]. In 2019, of the 1.9 billion women of reproductive age (15–49 years) globally; 1.1 billion had a need for family planning, that is, they were either current users of contraceptives or had unmet needs. Out of the proportion having the need or users of contraceptives; 842 million were using modern methods of contraception whilst 80 million were using traditional methods [2].

The use of contraceptives in sub-Saharan Africa (SSA) remains low [4]. For example, among the 252,962 women in reproductive age in 17 sub-Saharan African countries, only 17% used contraceptives. Among the preferred contraceptive methods, injectable was the most preferred (26%), followed by male condom and traditional methods 20% [4]. However, the use of injectable was 32% based on Multiple Indicator Cluster Surveys (MICS) in 20 African countries from 2013–2018 [5]. A similar study conducted by United Nations (2019) revealed that injectable accounts for 9.6% use in SSA. In Eastern and South-Eastern Asia, IUD is the most common contraceptive method used (18.6%), followed closely by male condom, 17% [2]. In Europe and Northern America, the pill and male condom are the most commonly used (17.8% and 14.6% of women, respectively), while in Latin America and the Caribbean, it is female sterilization and the pill (16.0% and 14.9% respectively). In Oceania, the dominant method is the pill (16.9%) and in Central and Southern Asia it is female sterilization (21.8% of women rely on this method). In Northern Africa and Western Asia, the two most common methods are the pill (10.5%) and IUD (9.5%) [2].

Factors that influence contraceptive use include; education, employment and communication with male partners [6]. Similarly, being sexually active, having children and being educated increase chances of using contraception [4, 5]. In addition, age, region of residence, marital status, religion, work status, higher education, middle to richest wealth index and high parity are associated with contraceptive use [7]. In the 2019 Multiple Indicator Cluster Survey, Ghana had 21% contraceptive use [5]. This prevalence was low compared to other countries within SSA. For instance, Zimbabwe has 62% of contraceptive use which is far higher than Ghana [5]. There could be certain factors that are associated with inequalities in contraceptive use in Ghana. This study aims at assessing the trend and inequalities of contraceptive use in Ghana based on the 1993–2014 Ghana Demographic and Health Surveys (GDHS). It is expected that the findings from this study can inform policy making and the design of interventions to accelerate Ghana’s trajectories towards the attainment of the Sustainable Development Goals (SDG) target 3.7, which seeks to ensure universal access to sexual and reproductive health-care services, including for family planning by 2030 [8].

## Methods

### Data source

Data from the 1993, 1998, 2003, 2008 and 2014 GDHS were analysed. GDHS forms part of the global surveys implemented by Measure Demographic and Health Survey (DHS) in about 85 low-and middle-income countries worldwide. The overarching focus of DHS is to collate information on children, women and men. Among the cardinal issues captured are contraceptive use. When sampling, selection of enumeration areas (EAs) is the first step and takes cognisance of rural and urban locations of Ghana. This is ensued by household selection from the EAs. The complete sampling procedure has been elaborated in the final reports of the 1993, 1998, 2003, 2008 and 2014 GDHS. The included sample for this study constituted sexually active women who answered questions pertaining to contraceptive use.

### Variables of interest

The study’s outcome variable was whether or not women were using contraceptives. Women who reported using contraceptives were categorised as “1”, whilst those who do not use contraceptives were classified otherwise “0”. Five stratifiers were used to assess inequality in contraceptive use: age (15–19 years and 20–49 years), economic status measured by wealth index (poorest, poorer, middle, richer, richest), education (no education, primary, secondary/higher education), residence (rural, urban) and region of residence (Western, Central, Greater Accra, Volta, Eastern, Ashanti, Brong Ahafo, Northern, Upper West, Upper East). Wealth index is derived by employing Principal Component Analysis (PCA). Education is measured by highest level of formal education completed.

### Statistical analyses

We used the 2019 updated World Health Organization (WHO)’s Health Equity Assessment Toolkit (HEAT) version 3.1 software for all analyses. Four measures were used to compute inequality namely Difference (D), Population Attributable risk (PAR), Population Attributable Fraction (PAF) and Ratio (R). Two of these are simple unweighted measures (D, R) and two are complex weighted measures (PAR, PAF). At the same time, R and PAF are relative measures whereas D and PAR are absolute measures. Summary measures were considered because WHO has indicated that both absolute and relative summary measures are essential for generating policy-driven findings [9, 10]. Unlike simple measures, the complex ones take size of categories inherent in a sub-population into account. WHO has extensively elaborated the procedure for generating summary measures.

In calculating D in age, we deducted contraceptive use among women aged 15–19 from those aged 20–49. For economic status, we deducted contraceptive use among the poorest group from contraceptive use in the richest group. For education, we computed D as contraceptive use among women with “no formal education” minus contraceptive use in “secondary/higher education”, while D in residence was about inequality between rural and urban residents. For region, D was computed by deducting the region with the lowest estimate from the region with the highest estimate. We computed the R for the variables with ordered responses such as education and wealth index as the difference between the most-disadvantaged sub-group (lowest quintile and uneducated) and the most-advantaged sub-group (highest quintile and secondary/higher education). We derived PAR by computing the difference in contraceptive use estimate of the reference category (*yref*) and overall average of the prevalence of contraceptive use. For the ordered variables, *y*_*ref*_ referred to the most-advantaged subgroups. In the case of age, residence, and region, which were non-ordered, y_ref_ meant the age, residence or region with the lowest estimate. The PAF was obtained by distributing PAR by the overall average μ, and further multiplied by 100 (PAF = [PAR / μ] * 100). Zero (0) PAF or PAR means no inequality, whilst a higher value indicates a relatively higher inequality. Variation in contraceptive use over the period was explored by making reference to the 95% uncertainty intervals (UIs) of the survey years. The absence of overlap in the UI means that a statistically significant difference existed between the UIs and vice versa.

### Ethical approval

The analyses were done using the publicly available Health Equity Assessment Toolkit (HEAT) software. HEAT contains the disaggregated DHS data that are publicly available via the WHO Health Equity Monitor database. Since ethical clearance was approved by the institution that commissioned, funded and managed the overall DHS program, further ethical clearance was not required. All GDHS survey data are freely available to the public. All surveys were approved by Inner City Fund international and the Ghana Health Service. The Measure DHS Program also ensured that the survey protocols complied with the U.S. Department of Health and Human Services regulations for protection of human subjects.

## Results

### Trends in contraceptive prevalence—use of modern and traditional methods in Ghana by different inequality dimensions, 1993–2014

Between 1993 and 2014, there was a steady increase in contraceptive prevalence from 20.3% to 26.7%. The contraceptive prevalence among women aged 20–49 increased from 20.6% [95% UI = 19.1, 22.3] in 1993 to 26.8% [95% UI = 24.9, 28.9] in 2014 and this exceeded the increase that was recorded among those aged 15–19 (from 13% [95 UI = 8.7, 19] to 18.6 [95% UI = 11.5, 28.6]), in the same period. Throughout the period studied, those in higher wealth quintile seemed to have higher prevalence of contraception use compared with the poor. Thus, contraceptive prevalence was relatively low among the women in poorest wealth quintile (11.8%, 12.3%, 14.0%, 14.2%, and 22.1% in 1993, 1998, 2003, 2008 and 2014, correspondingly). In the case of richest women, the contraceptive prevalence for those years were as follows: 34.2%, 33.0%, 34.6%, 31.4% and 28.0%. Regarding education, the contraceptive prevalence over the period was predominantly higher among women with at least secondary education. For instance, in 2014 those with secondary education or higher had 30.1% [95% UI = 27.4, 33] prevalence as compared with 18.6% [95% *UI* = 16.3, 21] among those with no education. The same trend was observed in the previous surveys. On residence, the analysis revealed that urban residents had higher contraceptive prevalence in 1993 (30.6% [95% UI = 27.7, 33.7]) than the rural residents (15.4% [95% UI = 13.7, 17.2]). However, an inverse observation was made in 2014 where the contraceptive prevalence among the rural residents (27.5% [95% UI = 25, 30.2]) exceeded the prevalence among the urban residents (25.8% [95% = 23, 28.8]). Across the regions, women in Greater Accra region dominated in contraceptive prevalence with 36.8% (95% UI = 30.3, 43.8) in 1993 whilst those in the Upper West region had the least prevalence (6.6% [95% UI = 2.4, 16.9]). This varied in 2014, such that Volta Region dominated in contraceptive prevalence (32.2% [95% UI = 26.8, 38.2]), whilst Northern Region recorded the lowest prevalence (11.2% [95% UI = 8.6, 14.5]) (Table 1).

**Table 1:** Trends in contraceptive prevalence in Ghana by different inequality dimensions, 1993–2014

**Dimension**	1993 (20.3)		1998 (22.0)		2003 (25.2)		2008 (23.5)		2014 (26.7)	
	**Sample (n)**	**1993 (20.3)**	**Sample (n)**	**1998 (22)**	**Sample (n)**	**2003 (25.2)**	**Sample (n)**	**2008 (23.5)**	**Sample (n)**	**2014 (26.7)**
		**% [95%** UI]		**% [95%** UI]		**% [95%** UI]		**% [95%** UI]		**% [95%** UI]
**Age**										
15–19 years	161	13[8.7, 19]	122.1	19.2[12.7, 28]	137.2	8.4[5, 13.9]	85	13.6[7.4, 23.8]	104.1	18.6[11.5, 28.6]
20–49 years	3043	20.6[19.1, 22.3]	3009	22.1[20.5, 23.8]	3411.7	25.8[24, 27.8]	2791.3	23.8[21.8, 25.9]	5217.4	26.8[24.9, 28.9]
**Economic Status**										
Poorest	541	11.8[9, 15.4]	731.1	12.3[10.1, 15.1]	753	14[11.5, 17]	572.8	14.2[10.9, 18.3]	1016	22.1[19.1, 25.3]
Poorer	657	11.4[9.4, 13.8]	582.9	15.9[13, 19.2]	687.4	24[20, 28.5]	576.9	20.3[16.7, 24.6]	964.2	27.2[23.5, 31.4]
Middle	688	17.3[14.7, 20.2]	565.6	22.3[18.9, 26.1]	692.5	24.9[20.8, 29.5]	525.2	21.8[18.1, 26]	1001.2	26.8[23.4, 30.4]
Richer	642	24.9[21.7, 28.5]	589.8	27.2[23.5, 31.2]	694.9	29[24.6, 33.8]	600.2	29[25, 33.4]	1089.8	28.9[25.8, 32.2]
Richest	676	34.2[30.7, 37.8]	661.7	33[28.7, 37.7]	721.1	34.6[31, 38.3]	601.2	31.4[26.6, 36.7]	1250.3	28[23.1, 33.5]
**Education**										
No education	1356	8.2[6.9, 9.7]	1105.9	13.2[11.1, 15.6]	1353.6	15.3[13.2, 17.6]	852.8	13.6[11.1, 16.4]	1478.2	18.6[16.3,21]
Primary school	1608	26.2[24, 28.6]	575.8	20.3[17,24]	710.4	26.1[22.2, 30.3]	638	26.6[22.9, 30.8]	978.7	28.9[25.4, 32.6]
*Secondary/higher education*	240	48.3[42.7, 54]	1449.3	29.3[26.8, 32]	1484.9	33.8[31.1, 36.6]	1385.5	28.2[25.5, 31.1]	2864.6	30.1[27.4, 33]
**Place of residence**										
Rural	2179	15.4[13.7, 17.2]	2152.9	18.1[16.4, 20]	2112.8	20.9[18.8, 23.3]	1660.3	20.9[18.4, 23.6]	2657.4	27.5[25, 30.2]
Urban	1025	30.6[27.7, 33.7]	978.2	30.4[27.3, 33.7]	1436.1	31.4[28.4, 34.6]	1216	27.1[24, 30.4]	2664.1	25.8[23, 28.8]
**Region**										
Ashanti	553	13.7[11.6, 16.2]	491	24.6[20.3, 29.5]	643.2	29.7[25, 34.9]	542.2	27[22.8, 31.7]	968.7	26.4[21.9, 31.6]
Brong Ahafo	307	25.4[21.2, 30.2]	234.6	24.7[20.4, 29.6]	398.4	33[27.8, 38.5]	267.2	29[23.1, 35.6]	438.9	30.1[25.1, 35.7]
Centre	301	15.6[12.2, 19.8]	338.3	19.3[14.6, 25.1]	274.4	15.2[10.7, 21]	254.2	22.9[16.3, 31.2]	531.7	31.1[27.2, 35.2]
East	340	25.9[20.8, 31.8]	425.6	26.6[21.6, 32.4]	354.1	27.1[21.6, 33.3]	252.2	24.2[18.6, 30.9]	500.3	29.4[24.6, 34.6]
Greater Accra	356	36.8[30.3, 43.8]	449.4	32.2[28, 36.7]	475.9	34[28.5, 39.9]	422.2	32.6[26.6, 39.3]	1005.3	28.7[22.8, 35.5]
Northern	376	11.2[8, 15.4]	195.6	10[6.5, 15.1]	431	12.1[9.6, 15.3]	337.7	5.9[3.5, 9.8]	560.7	11.2[8.6, 14.5]
Upper East	236	10.2[5.3, 18.5]	97.5	11.9[7.1, 19.2]	236.3	11.9[62.2, 21.5]	168.1	14.7[9.2, 22.5]	218.2	23.7[19.9, 28]
Upper West	136	6.6[2.4, 16.9]	209.4	9[6.3, 12.6]	113.3	26.3[22.3, 30.8]	81.5	21.7[17.3, 26.8]	145.9	25.2[20.4, 30.8]
Volta	349	25.2[21, 29.9]	334	21.1[16.9, 26.1]	303.6	23.6[18.9, 29]	290.2	28.6[21.7, 36.8]	404.9	32.2[26.8, 38.2]
Western	250	26.4[20.1, 33.9]	355.5	18.3[14.6, 22.7]	318.7	28.2[23.5, 33.5]	260.8	19.1[14.4, 24.9]	546.8	27.1[22.1, 32.8]

### Inequality indices estimates of the factors associated with contraceptive prevalence – use of modern and traditional methods in Ghana

**Fig. 1:**
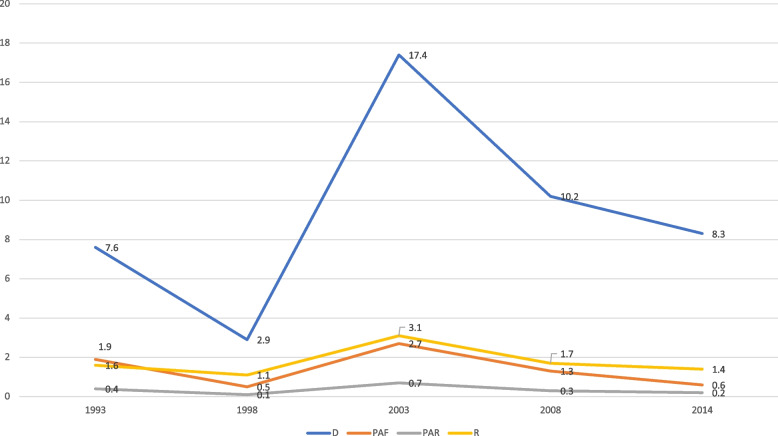
Age-based inequality in contraceptive prevalence in Ghana from 1993 to 2014

**Fig. 2:**
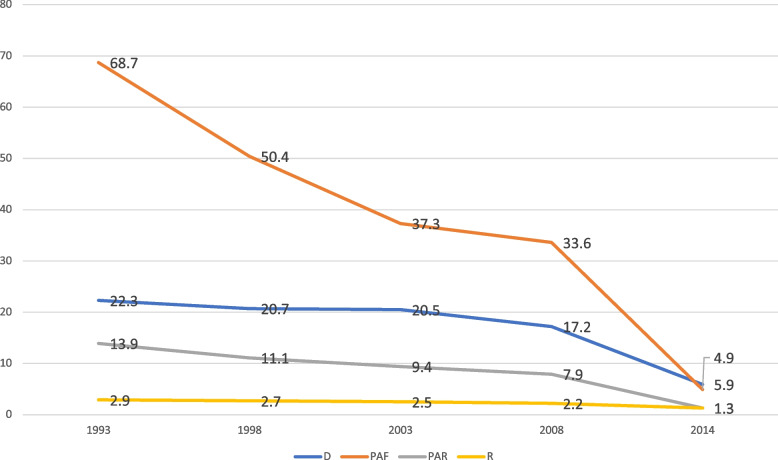
Economic status-based inequality in contraceptive prevalence in Ghana from 1993 to 2014

**Fig. 3:**
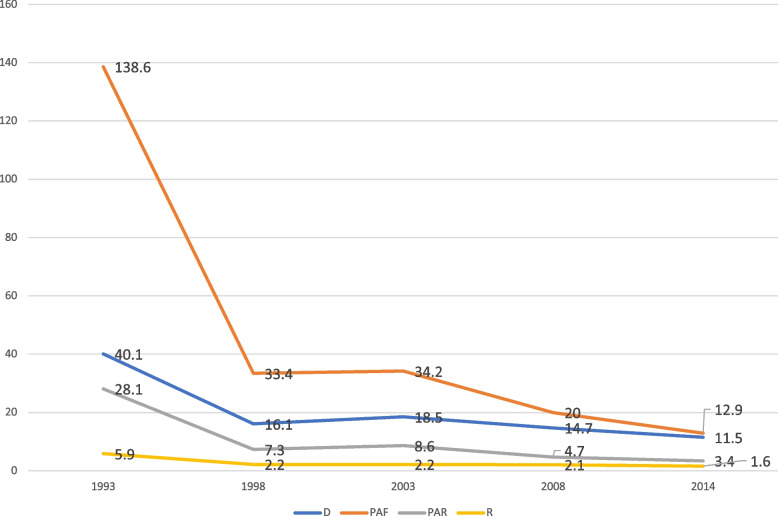
Education-based inequality in contraceptive prevalence in Ghana from 1993 to 2014

**Fig. 4:**
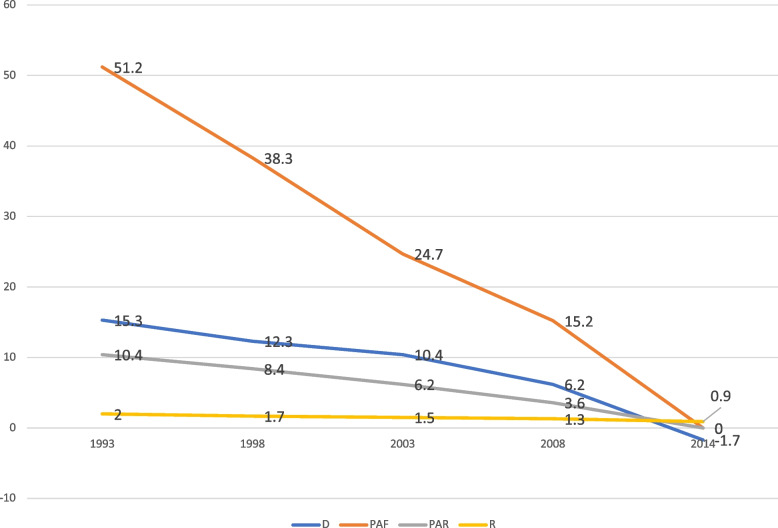
Residence-based inequality in contraceptive prevalence in Ghana from 1993 to 2014

**Fig. 5:**
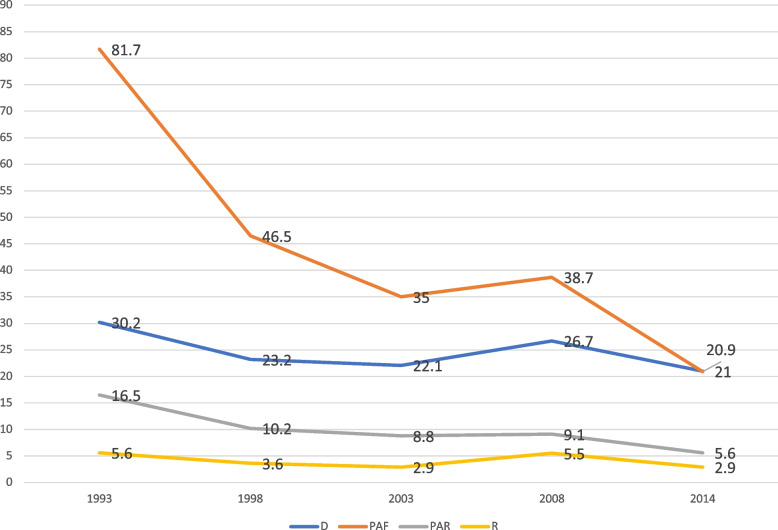
Regional-based inequality in contraceptive prevalence in Ghana from 1993 to 2014

The analysis showed considerable variation in contraceptive use over the period studied, thus 1993 to 2014. This reflected in both the simple (D and R) and complex (PAF and PAR) measures, as illustrated in Figs. 1, 2, 3, 4 and 5. On age, it was evident that substantial inequality existed with respect to contraceptive use, from 1993 to 2014, with widest inequality occurring in 2003. This manifested in the PAF (PAF = 2.7, 95% UI = -16.6–21.9) and the D (D = 17.4, 95% UI = 12.7–22.1) (Fig. 1). Contraceptive use inequality also occurred in terms of economic status of the women, meanwhile, the gap appeared to be closing with time. As such, the least inequality was observed in 2014 with PAR (PAR = 1.3, 95% UI = -1–3.6) and D (D = 5.9, 95% UI = -0.1–12) measures (Fig. 2). Regarding education, the widest inequality occurred in 1993, manifesting in the PAF (PAF = 138.6, UI = 132.1–145.1) and the D (D = 40.1, 95% UI = 34.4–45.9). Meanwhile, the least inequality was noted in 2014 (Fig. 3).

With reference to place of residence, it was similarly noted that the widest gap in inequality occurred in 1993, which reflected in the PAF (PAF = 51.2, 95% UI = 46.2–56.3) and D (D = 15.3, 95% UI = 11.8–18.7). It is also worthy to mention that no inequality in contraceptive use occurred in 2014, as far as residence is concerned (Fig. 4). Yet, there was absolute (D and PAR) and relative (R and PAF) inequality in contraception use with respect to sub-national Regions for the period. For instance, in the case of 2014, the D (D = 21, 95% UI = 14.6–27.4) and the PAF measure (PAF = 20.9, 95% UI 11.2 − 30.5) revealed substantial absolute and relative regional inequality between the region with the highest contraception use (Northern region) and the region with the least usage (i.e. Volta region) as shown in Fig. 5.

## Discussion

Contraceptive use is an undeniable pathway to reducing total fertility rate in any given population. Nevertheless, there is compelling evidence that suggests that disparities exist in Ghana in terms of access and utilization of contraceptives [4, 5]. We therefore sought to examine the trends of inequalities in the use of both traditional and modern contraceptives in Ghana.

Our study reveals that there was a steady increase in contraceptive prevalence from 20.3% in 1993 to 26.7% in 2014. Thus, suggesting a 6.4% change over the period. This increase in contraceptive use over the period is lower than what has been reported in Kenya, where there was a 77.5% change increase in the contraceptive use prevalence between 1989 and 2014 [11]. However, the rate of change as observed in this study is higher when compared to Ethiopia, Niger and Nigeria that have reported lower percentage change in contraceptive use prevalence [12, 13]. The increasing trend in contraceptive use prevalence in Ghana is synonymous to findings from earlier studies [14, 15]. We postulate that the observed increasing trend in contraceptive use prevalence could be a ripple effect of massive contraceptive use campaigns championed across the country over the years.

An instance is the school health education programme (SHEP) that provides constant information about contraceptives and other reproductive health matters in pre-tertiary educational institutions [16]. Furthermore, Ghana’s contraceptive prevalence increase over the years could also be linked to the implementation of strategies from 2012 London Summit on Family Planning [12]. Ahmed et al. [12] posit that after the 2012 London Summit on Family Planning, Ghana experienced 3·64%-point increase in contraceptive use prevalence. Other initiatives such as the Community-based Health Planning Services (CHPS) may also have contributed to the increasing trend of contraceptive use prevalence in Ghana between 1993–2014 [15].

The results indicate that consistently, the prevalence of contraceptive use was higher among adults compared to adolescents (i.e., 15–19 years). Similar trend was reported by Appiah et al. [17]. A plausible explanation could be that, although Ghana has over the years increased contraceptive awareness, adolescents continue to face stigma in accessing modern contraceptives, while many are oblivious about traditional contraceptive methods [18]. This could account for the low prevalence of contraceptive use among this cohort. Another plausible explanation could be that unlike adolescents who may not have a regular sexual partner, hence making them relatively less sexually active, adults may have regular sexual partner. Hence, increasing the prevalence of contraceptive use among their cohort. Concerning the inequality trend, we realised that substantial inequality occurred in 2003 while the least inequality occurred in 2014. This is a clear indication of improvements made to increase contraceptive use among adolescents. The results may be explained from the point that in 2003, the number of adolescent-and-youth friendly facilities in the country was significantly low compared to the number of such facilities in 2014 [17].

We observed that between 1993 and 2014, contraceptive use prevalence was high among persons living in urban areas and among those residing in Greater Accra region. These findings are in alignment with earlier studies conducted in Ghana [15, 19] and Ethiopia [20]. With the concentration of government initiatives and interventions in urban areas, particularly in Greater Accra which harbors the country’s capital, it was not surprising that the contraceptive use prevalence was consistently high in these areas. In terms of place of residence, the study shows that the widest gap in inequality occurred in 1993. However, there was no inequality in contraceptive use in 2014. One possible explanation for this observation could be the proliferation of CHPS facilities across all regions in the country. As such, rural residences which hitherto did not have health facilities are currently near healthcare facilities where they can access information on contraceptives. Across the survey points, it was observed that those with secondary or higher education had the highest prevalence of contraceptive use. This affirms Aviisah et al.’s [15] finding that the trend of contraceptive use prevalence was high among those with higher educational attainment compared to those with no formal education.

For wealth quintile, our study shows that over the period 1993–2014, those in the richest quintile consistently reported higher contraceptive use prevalence. Our findings contradict Aviisah et al.’s [15] finding that found trends of low contraceptive use prevalence among women in the richest quintile. Our result is in-congruent with findings from an Ethiopian [20] and Nigerian study [21]. However, the findings align with a study from Brazil [22]. Often, women in the richest quintile have the financial resources and capacity to easily afford reproductive health services including access and use of modern contraceptives [23]. The findings of this study also indicate that income-related inequalities in connection to contraceptive use has reduced; a result that is synonymous to the findings of a study by Asamoah et al. [14]. In Creanga et al.’s [24] perspective, women in the richest wealth quintile are more likely to use long-term methods of contraception which are often expensive. Thus, higher income suggests greater use of contraceptives. This could explain why income inequality in current contraceptive use is diminishing.

### Policy implications

Although there is an increasing trend in contraceptive use prevalence, the percentage change is lower than expected, with substantial educational, regional, wealth and regional variations. Therefore, policymakers must focus on increasing secondary and higher education in the country as well as expanding women’s economic opportunities. This may significantly lead to lower inequalities in contraceptive use. As our findings indicate an improving status quo in relation to income-related inequalities in contraceptive use, there is the need for the government to strengthen its job creation strategies and programs.

### Strengths and limitations

The nature of the GDHS precludes us from making causal inferences, and that is a huge limitation. Another important limitation of this study is the dataset used. The latest GDHS is the 2014 survey. However, we acknowledge that between 2014 and now, there may have been some changes and as such, the findings might not necessarily reflect the current status quo. We, however, do not expect drastic deviation from the status quo. Notwithstanding, the use of both simple and complex measures of inequality contributes to the quality of our results as the limitations of each group of measure is dealt with by the strengths of the others. Also, the use of WHO’s HEAT software for the analysis confirms the reliability of the findings.

## Conclusion

We conclude that there was a steady increase in contraceptive use prevalence from 20.3% in 1993 to 26.7% in 2014. Nevertheless, the percentage change is minimal. The trends of inequality indicate that inequalities in contraceptive use was evident across the dimension of age, place of residence, wealth index, education and region. Yet, there was substantial reduction in inequalities related to contraceptive use in 2014. Therefore, targeting adolescents, women in rural areas, those in low wealth quintile and those with no formal education is invaluable to substantially overcome the inequality in contraceptive use across the country.

## Data Availability

The datasets generated and/or analyzed during the current study are available in the WHO’s HEAT version 3.1 [https://www.who.int/gho/health_equity/assessment_toolkit/en/].

## Abbreviations

CHPS: Community-based Health Planning Services
EAs: Enumeration Areas
GDHS: Ghana Demographic and Health Surveys
PAF: Population Attributable Fraction
PAR: Population Attributable Risk
PCA: Principal Component Analysis
SDG: Sustainable Development Goals
SHEP: School health education programme
SSA: Sub-Saharan Africa
